# Influence of Blood Components on Neuroinflammation, Blood–Brain Barrier Breakdown, and Functional Damage After Acute Subdural Hematoma in Rats

**DOI:** 10.1089/neur.2023.0098

**Published:** 2024-03-06

**Authors:** Daniel Jussen, Syamend Saeed, Tatjana Jablonski, Harald Krenzlin, Kristin Lucia, Tobias Kraemer, Oliver Kempski, Marcus Czabanka, Florian Ringel, Beat Alessandri

**Affiliations:** ^1^ Department of Neurosurgery, Goethe University, Frankfurt am Main, Germany.; ^2^ Johannes Gutenberg University, Institute for Neurosurgical Pathophysiology, Mainz, Germany.; ^3^ Department of Neurosurgery, University Medical Center Mainz, Mainz, Germany.

**Keywords:** acute subdural hematoma, blood–brain barrier, blood constituents, cerebral blood flow, inflammation, rat

## Abstract

A central component of injury development after acute subdural hematoma (ASDH) is the increased intracranial pressure and consecutive mechanical reduction of cerebral blood flow (CBF). However, the role of different blood constituents in ASDH as additional lesioning factors remains unclear. This study examines the influence of blood components on neuroinflammation, blood–brain barrier (BBB) breakdown, and functional deficits in a rat model of ASDH. We infused corpuscular (whole blood, whole blood lysate, and red cell blood) and plasmatic (blood plasma, anticoagulated blood plasma, and aqueous isotonic solution) blood components into the subdural space while CBF was monitored. Rats then underwent behavioral testing. Lesion analysis and immunohistochemistry were performed 2 days after ASDH. Inflammatory reaction was assessed using staining for ionized calcium-binding adaptor molecule 1 and glial fibrillary acidic protein, interleukin-1ß, tumor necrosis factor-alpha, and membrane attack complex. Integrity of the BBB was evaluated with albumin and matrix metalloproteinase 9 (MMP9) staining. We observed a significant drop in CBF in the corpuscular group (75% ± 7.5% of baseline) with distinct post-operative deficits and larger lesion volume compared to the plasmatic group (13.6 ± 5.4 vs. 1.3 ± 0.4 mm^3^). Further, inflammation was significantly increased in the corpuscular group with stronger immunoreaction. After whole blood infusion, albumin and MMP9 immunoreaction were significantly increased, pointing toward a disrupted BBB. The interaction between corpuscular and plasmatic blood components seems to be a key factor in the detrimental impact of ASDH. This interaction results in neuroinflammation and BBB leakage. These findings underscore the importance of performing surgery as early as possible and also provide indications for potential pharmacological targets.

## Introduction

The severe consequences of acute subdural hematoma (ASDH) after traumatic brain injury (TBI) have been demonstrated extensively by previous studies.^1,2^ Treatment consists of surgical evacuation with or without decompressive hemicraniectomy.^3^ Medical considerations involve the prevention and handling of seizures, along with the management and restarting of antithrombotic and -coagulant medications. In the experimental setting, blood clot evacuation leads to improved intracranial pressure (ICP), cerebral blood flow (CBF), and consecutively to the reduction of cerebral lesions.^4,5^ The positive effect of surgery is partially attributable to the resulting improved cerebral hemodynamics, tissue oxygenation, and reduced ICP, which are associated with better functional outcomes.^6,7^ Further, blood constituents themselves may play a significant role in lesion development; however, basic research on this aspect remains sparse. Blood-constituent–triggered pathomechanisms have been shown to lead to early reduction of glucose metabolism followed by more severe edema and histological damage.^8^

Recent data have found differences in lesion size, inflammatory reaction, and behavioral deficits after either subdural blood or liquid paraffin infusion.^9^ Despite comparable ICP, cerebral perfusion pressure, and CBF, time-dependent lesion growth and more severe neurological deficits were observed in the blood infusion groups. Given that blood is composed of cells suspended in plasma, each including various proteins, electrolytes, etc., it remains unclear which of these blood constituents are responsible for secondary brain damage. Studies of spreading ischemia have shown that products of hemolysis, such as K^+^ and hemoglobin (Hb), lead to widespread necrosis of the cortex.^10^

Further, it has been demonstrated that heme products play a central role in injury after intracranial hemorrhage (ICH).^11^ Erythrocyte components, such as methemoglobin, in the subarachnoid space induce microglial activation and tumor necrosis factor (TNF) upregulation by Toll-like receptor 4, promoting widespread neuroinflammation.^12^ Whereas plasmatic constituents such as thrombin contribute to secondary brain injury,^13–15^ intracellular components such as carbonic anhydrase 1 (CA-1) from lysed erythrocytes have also been shown to induce edema and lead to microglial activation and neuronal death.^16^ We therefore examined the influence of different blood constituents on lesion size, neuroinflammation, blood–brain barrier (BBB) disruption, and functional outcome after acute subdural hematoma in rats. We hypothesized a multi-factorial role of blood constituents on the pathophysiology after ASDH with both intracellular factors (such as Hb, CA-1, and K^+^) and plasmatic factors such as thrombin causing damage. To this end, the influence of corpuscular components was examined in a whole blood and red cell concentrate subdural infusion group. To differentiate between intracellular and membrane constituents, we included a group with lysed whole blood to determine the effect of intracellular components. Further, we focused on plasmatic features, including a group inhibiting thrombin and an artificial fluid resembling the electrolyte composition of blood plasma.

## Methods

### Subjects

All experiments were performed under the Animal Care and Welfare Guidelines and were approved by the local ethics committee. Male Sprague-Dawley rats weighing 296–350 g (Charles River, Sulzfeld, Germany) were used for this experiment (*n* = 62).

### Experimental groups

Animals were randomly assigned to the corpuscular or plasmatic group. For further analysis, the following subgroups containing either corpuscular blood components were established: whole blood (*n* = 10), whole blood lysate (*n* = 11), and red cell concentrate (*n* = 9) or plasmatic blood constituents: blood plasma (*n* = 11), anticoagulated plasma (*n* = 11), and aqueous isotonic solution (*n* = 10).

Blood was obtained from a jugular vein catheter. In the whole blood group, 300 μL of autologous venous blood was infused subdurally. This group was used as a control group for corpuscular constituents. In the whole blood lysate group, venous blood was lysed using ultrasound (Sonopuls HD70; Bandeln Electronics, Berlin, Germany) for 30 sec before subdural infusion. In the red cell concentrate group, venous blood was centrifuged for 2 min at 8000 RPM/*g* (Denver Instrument Company, Arvada, CO). Afterward, blood plasma was removed and replaced with an aqueous isotonic solution (see below). Venous blood was centrifuged for 2 min for the blood plasma group, and 300 μL of plasma was decanted. This group was used as a control group for plasmatic constituents. In the anticoagulated blood group, whole blood was mixed with 10 μL of small molecule direct thrombin inhibitor argatroban (1:100; Mitsubishi Tanabe Pharma, Osaka, Japan), centrifuged, and the plasma was used for subdural infusion. The aqueous isotonic solution consisted of ions in 1 L of distilled water, pH 7.4, temperature 37°C: KCl = 4 mmol/L, NaCl = 105 mmol/L, CaCl_2_ = 1 mmol/L, MgCl_2_ = 1 mmol/L, and NHCO_3_^-^ = 30 mmol/L.

### Surgical preparation

**FIG. 1.**
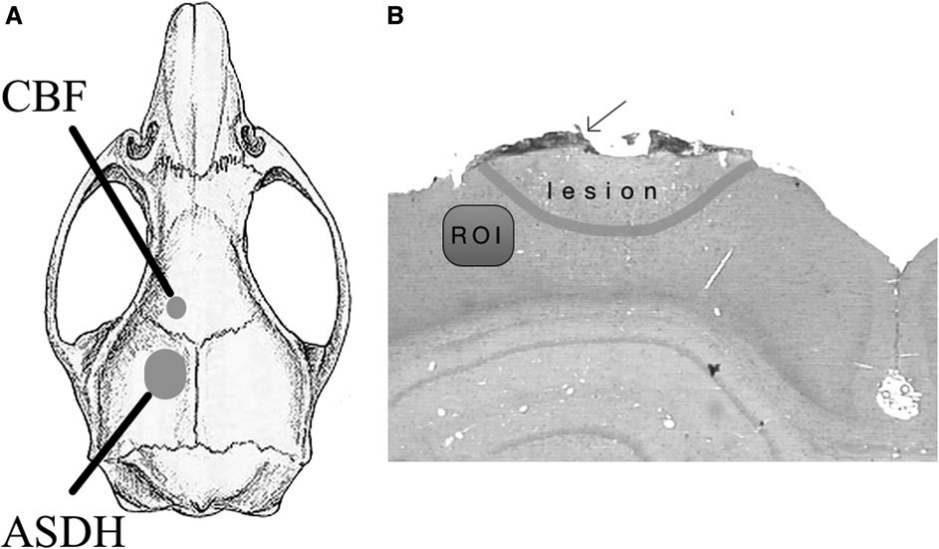
(**A**) Schematic drawing showing the site of ASDH infusion and CBF measurement. (**B**) Coronal section of hematoxylin and eosin staining for neurohistological evaluation; arrow: residual subdural ASDH. ASDH, acute subdural hematoma; CBF, cerebral blood flow; ROI, region of interest.

Animals were anesthetized with chloral hydrate (36 mg/mL) using 1 mL/100 g body weight as an initial dose (intraperitoneally) and 1 mL/h to maintain anesthesia with an intraperitoneal catheter. Each animal was injected with 1 mg of atropine. Body temperature was constant at 37°C with a feedback-controlled homeothermic blanket (Harvard, South Natick, MA). Rats were intubated and mechanically ventilated. A polyethylene tube was inserted into the tail artery for arterial blood pressure monitoring and blood gas analysis. The volume needed for each blood gas analysis was 210 μL (ABL615/EML105; Radiometer, Copenhagen, Denmark). A jugular vein was cannulated to obtain blood for subdural infusion. Rats were fixed in a stereotaxic frame. After craniotomy, 1 mm lateral to the midline and 1 mm posterior to the bregma, a 23-gauge L-shaped blunted needle (Sterican; B. Braun, Melsungen, Germany) was inserted under the dura mater, and the craniotomy was sealed again with tissue glue (Histoacryl; B. Braun). Anterior to the infusion needle and coronal suture, a skull area of 2 × 2 mm was thinned out. A laser-Doppler probe (Moor Instruments Ltd, Axminster, UK) was placed over this window to measure CBF (Fig. 1). After an equilibration period with stable physiological values, a 10-min baseline period was acquired. Next, 300 μL of the above-described blood components were subdurally infused at a 50-μL/min rate.^17^ Animals were monitored for 40 min from this time onward.

### Behavioral evaluation

Neurological and behavioral testing were performed in a quiet room in dim light. Starting 72 h before the operation, all animals were trained daily to walk along a beam (diameter 1.8 cm, length 1 m, 2.5 cm between bars, and 50 cm above ground) with the time it took to reach a black box being recorded.^18^ Balance was assessed using a beam balance test for 60 sec, graded on a 3-point scale: 1 point for 180-degree rotation; 1 point for balancing with steady posture; and 1 point for not hugging the beam with limbs or hanging freely.^19^ Sensory and motor integrity were tested using a neuroscore by evaluating motor activity, orientation, and reaction to tactile, visual, and auditory stimuli. The points obtained in the individual tests were then added to yield an overall score ranging from 0 points (no deficit) to 60 points (most severe deficit).^4^ Twenty-four hours before the operation, baseline measures were performed, and tests were repeated 48 h after the operation.

### Neurohistological evaluation

Animals were perfused transcardially with 4% buffered paraformaldehyde at 48 h after ASDH, and their brains were carefully removed and post-fixed for 24 h. Coronal sections of 5-μm slice thickness spaced 250 μm apart were made through the paraffin-embedded brains and stained with hematoxylin and eosin to delineate the injury. The damaged area on each section was photographed with a CCD camera (SSC-C370P; Sony Corporation, Tokyo, Japan) connected to a light microscope (Axiopod 2; Zeiss, Oberkochen, Germany). The areas of brain damage in the traumatized hemisphere were surveyed with image-analyzing software (Optimas 6.51; VSG, UK).

Sections were immunohistologically stained for glial fibrillary acidic protein (GFAP; astrocytes, 1:300; BD Biosciences, San Jose, CA), ionizing calcium-binding adaptor molecule 1 (Iba1; microglia; 1:300; Wako Chemicals USA, Richmond, VA), TNF-α (1:100; Santa Cruz Biotechnology, Santa cruz, CA), matrix metalloprotease 9 (MMP9; 1:100; Abcam, Cambridge, MA), interleukin-1ß (IL-1ß; 1:20; Neuromics, Edina, MN), membrane attack complex (MAC; 1:20; LifeSpan Bio Sciences, Shirley, MA), and albumin (BBB destruction; 1:300, NBP1-32458; Novus Biologicals, Centennial, CO).

Sections were deparaffinated with Xylol and, after descending alcohol series, treated with 2% hydrogen peroxide and incubated with 5% normal horse serum for GFAP and MAC. IL-1ß with 5% rabbit and albumin, Iba1, TNF-α, and MMP9 with 5% goat serum. The primary antibody was incubated overnight at 4°C. Subsequently, slides were rinsed in phosphate-buffered saline and treated with the second antibody for 30 min. The reaction product was visualized with diaminobenzidine tetrahydrochloride for 3 min (Vector Laboratories, Newark, CA). Immunoreactive areas were calculated in a region of interest (ROI; size, 150*300 μm) directly adjacent to the contusion on the ipsilateral hemisphere (ROI; Fig. 1).

### Microglia activation

Morphometric analysis of Iba1-positive cells was performed to characterize microglia reactivity using ImageJ Processing and Analysis Software (V1.52a; National Institutes of Health, Bethesda, MD). Mean circularity (4π*(area/circumference^2^)) and roundness (4*area/(π*major axis^2^) for 10 cells within the ROI were determined. Higher values indicate a rounder and thus activated cell, with lower values indicating a ramified resting cell.

### Statistical analysis

Data are expressed as mean ± standard error of the mean (SEM). Groups were compared with a one-way analysis of variance with *post hoc* comparisons for individual differences (Student-Newman-Keuls' test). Data that were not distributed normally were analyzed by Kruskal-Wallis' analysis of variance on ranks followed by Dunn's *post hoc* test. Repeated-measures analysis of variance was done for CBF comparison with baseline (Sigma-Plot 11.0; Systat, San Jose, CA). Differences were considered statistically significant at *p* < 0.05.

## Results

### Physiological variables

All blood parameters were within physiological ranges (Tables 1 and 2). None of the infused subdural volumes affected any analyzed parameters (not shown). Mean blood K^+^ concentrations were 5.4 ± 0.5 mmol/L (excluding values from lysed blood), whereas K^+^ concentrations in lysed venous whole blood preparation were 42.0 ± 0.9 mmol/L.

**Table 1.** Physiological Variables in Corpuscular Group

***Group***	***pH***	***pCO_2_ [mm Hg]***	***pO_2_ [mm Hg]***	***Hb [g/dl]***	***Hct [%]***	***Glu [mmol/L]***	***Lac [mmol/L]***
Whole blood	7.39 ± 0.10	45.4 ± 1.7	103.1 ± 5.0	13.23 ± 0.20	40.6 ± 0.7	332.4 ± 17.0	1.6 ± 0.1
Whole blood lysate	7.42 ± 0.00	41.6 ± 0.7	102.6 ± 4.2	13.1 ± 0.2	40.1 ± 0.6	351.7 ± 19.8	2.0 ± 0.1
Red cell blood	7.39 ± 0.10	46.6 ± 1.9	102.1 ± 5.6	13.2 ± 0.2	40.4 ± 0.7	319.8 ± 16.4	1.5 ± 0.1

Hb, hemoglobin; Hct, hematocrit; Glu, glucose; Lac, lactate.

**Table 2.** Physiological Variables in Plasmatic Group

***Group***	***pH***	***pCO_2_ [mm Hg]***	***pO_2_ [mm Hg]***	***Hb [g/dL]***	***Hct [%]***	***Glu [mmol/L]***	***Lac [mmol/L]***
Blood plasma	7.42 ± 0.10	41.9 ± 1.4	103.3 ± 5.1	13.3 ± 0.2	40.9 ± 0.6	332.3 ± 21.0	1.6 ± 0.1
Anticoagulated blood plasma	7.41 ± 0.10	44.1 ± 2.1	109.3 ± 5.5	13.2 ± 0.2	40.6 ± 0.6	333.4 ± 37.8	1.6 ± 0.1
Aqueous isotonic solution	7.4 ± 0.1	42.5 ± 1.2	113.3 ± 7.1	13.3 ± 0.3	40.8 ± 0.9	362.9 ± 36.1	1.8 ± 0.3

Hb, hemoglobin; Hct, hematocrit; Glu, glucose; Lac, lactate.

### Effects of blood constituents on cerebral blood flow

**FIG. 2.**
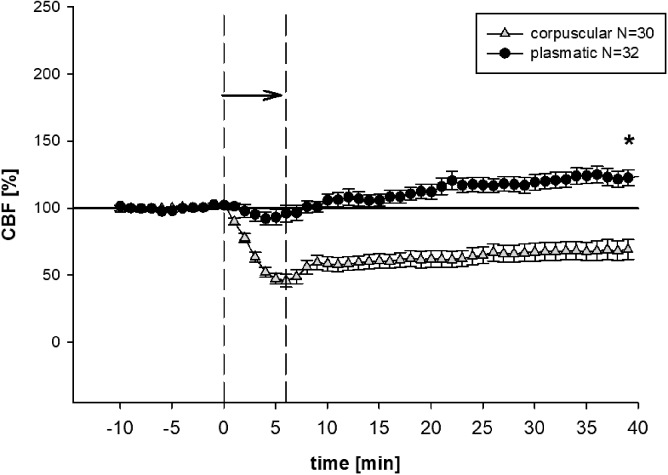
CBF. Mean (± SEM) CBF throughout the experimental procedure. The arrow indicates the time of subdural infusion. In the corpuscular group, subdural infusion lowered CBF, *p < 0.001 versus plasmatic group at the end of the operation. In the plasmatic group, after a transient drop, all groups exceeded the baseline values. CBF, cerebral blood flow; SEM, standard error of the mean.

Mean baseline values for CBF were 200 ± 20 arbitrary units. For purposes of clarity, all values were normalized to baseline (100%) and presented as change in percent. As such, CBF dropped significantly in the corpuscular group, most markedly with a minimum of 6 min after the start of infusion. CBF was reduced to 75% ± 7.5% in the corpuscular group 40 min after infusion (*p* < 0.001). There was only a temporary decline in CBF in the plasmatic group (Fig. 2).

### Effects of blood constituents on neurological and motor deficits

**FIG. 3.**
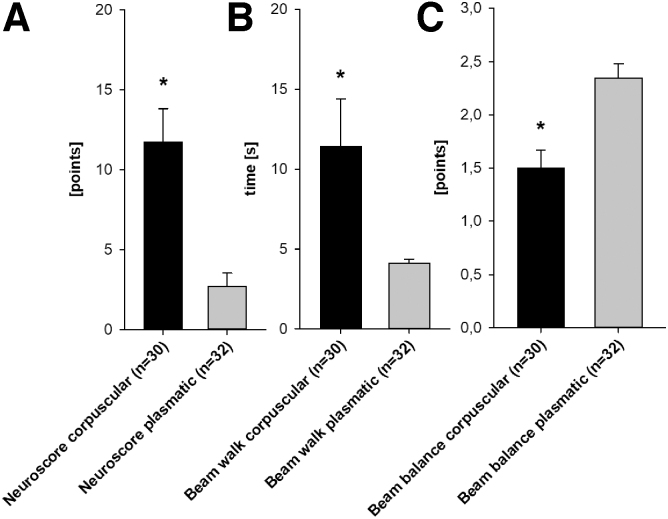
Behavioral evaluation. Corpuscular groups had significantly worse performance in (**A**) neuroscore, (**B**) beam-walk, and (**C**) beam balance tests (mean ± SEM); *p < 0.05 versus plasmatic group. SEM, standard error of the mean.

All rats had a pre-operative neuroscore of 0 points (indicating no deficit). The corpuscular group showed distinct neurological deficits in the post-operative evaluation, whereas the plasmatic group showed only minor deficits without baseline differences. There was a significant post-operative difference between both groups (corpuscular group 11.73 ± 2.10 points vs. plasmatic group 2.7 ± 0.8, *p* < 0.001; Fig. 3).

All rats were able to pass the beam-walk test after the pre-operative training period in under 10 seconds. Forty-eight hours after surgery, corpuscular group animals needed significantly more time to pass the test. In contrast, plasmatic group animals maintained baseline levels (corpuscular group 11.4 ± 3.0 vs. plasmatic group 4.1 ± 0.2 sec, *p* < 0.001; Fig. 3).

The beam balance test could also be performed by all rats pre-operatively. After surgery, corpuscular animals had fewer points on the 3-point scale (indicating a higher deficit) when compared to the plasmatic group (corpuscular group 1.5 ± 0.2 points vs. plasmatic group 2.3 ± 0.1 points, *p* < 0.001; Fig. 3).

### Histology

**FIG. 4.**
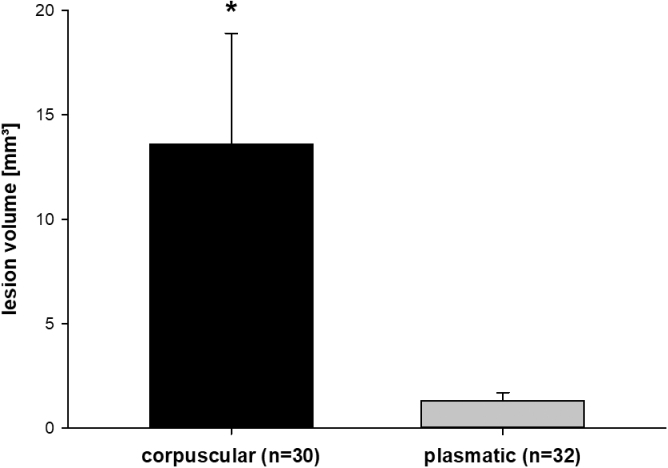
Lesion volume. Lesion volume (mean ± SEM) induced by subdural infusion of 300 μL of different blood components. The corpuscular group had larger lesion volumes compared to the plasmatic group; *p < 0.05. SEM, standard error of the mean.

The corpuscular group had significantly larger lesion volumes than the plasmatic group (corpuscular group 13.6 ± 5.4 vs. plasmatic group 1.3 ± 0.4 mm^3^, *p* < 0.05; Fig. 4). Because the plasmatic group showed nearly no lesions (aqueous isotonic solution 0.5 ± 0.2, blood plasma 0.7 ± 0.1, and anticoagulated plasma 1.5 ± 1.0 mm^3^), further subgroup analysis was done within the corpuscular groups (whole blood, whole blood lysate, and red cell concentrate).

**FIG. 5.**
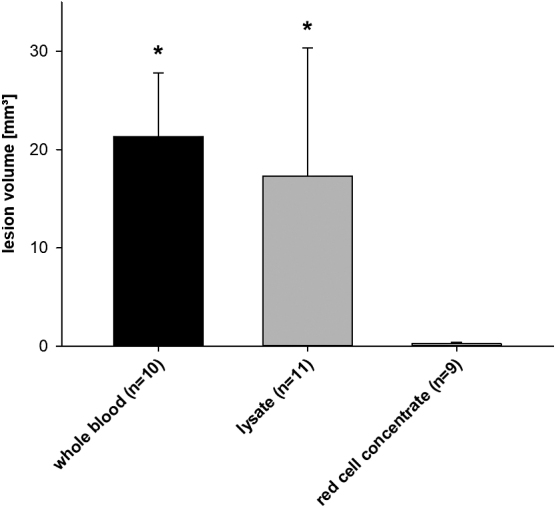
Lesion volume subgroups. Lesion volume (mean ± SEM) induced by subdural infusion of 300 μL of different corpuscular blood components. Whole and whole blood lysate had significantly larger lesion volumes compared to red cell concentrate; *p < 0.05. SEM, standard error of the mean.

Here, we observed significantly larger lesions in the whole blood groups (whole blood 21.3 ± 6.5, whole blood lysate 17.3 ± 13.0, and red cell concentrate 0.3 ± 0.1 mm^3^, *p* < 0.05 whole blood and whole blood lysate vs. red cell concentrate; Fig. 5).

### Inflammatory reaction

**FIG. 6.**
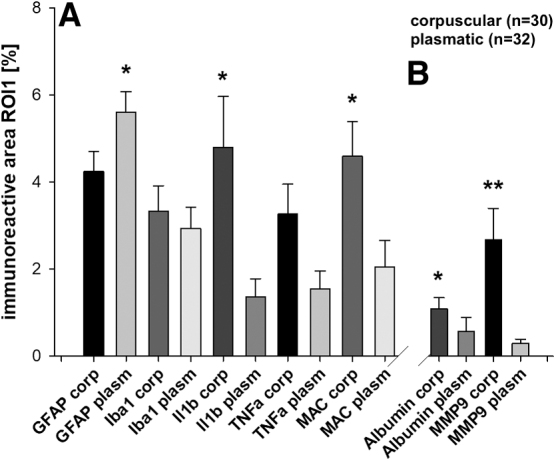
(**A**) Inflammatory reaction and (**B**) BBB function after ASDH. In the corpuscular group, there was higher immunoreactivity in IL1b and MAC after 48 h and signs for BBB disruption; *p < 0.05, **p < 0.001. Values are given as mean ± SEM. ASDH, acute subdural hematoma; BBB, blood–brain barrier; GFAP, glial fibrillary acidic protein; Iba1, ionized calcium-binding adaptor molecule 1; IL1b, interleukin-1ß; MAC, membrane attack complex; MMP9, matrix metalloproteinase 9; ROI, region of interest; SEM, standard error of the mean; TNFa, tumor necrosis factor-alpha.

In an overview comparing corpuscular and plasmatic groups, there were significantly higher immunoreactive areas in Il1b and MAC (IL1b corpuscular 4.8 ± 1.2 vs. plasmatic 1.4 ± 0.4%, *p* < 0.05 and MAC corpuscular 4.6 ± 0.8 vs. plasmatic 2.1 ± 0.6%, *p* < 0.05), but a higher GFAP immunoreactivity in the plasmatic group (corpuscular 4.3 ± 0.5 vs. plasmatic 5.6 ± 0.5%, *p* < 0.05; Fig. 6).

**FIG. 7.**
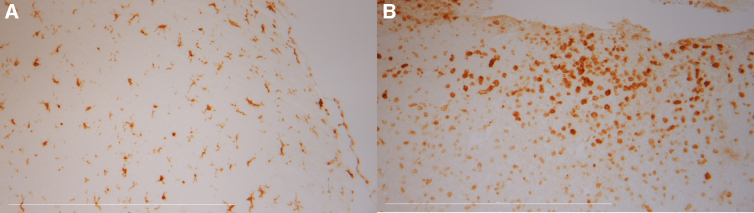
Microglia morphology. Representative section of a whole blood animal showing (**A**) ramified (resting) in the contralateral and (**B**) round (activated) microglia in the ipsilateral hemisphere.

There were no differences in Iba1 immunoreactivity, but when looking at microglia morphology (Fig. 7), we found more activated microglia in the corpuscular group when compared to the plasmatic groups (roundness corpuscular 0.70 ± 0.02 vs. plasmatic groups 0.60 ± 0.02, *p* < 0.05, circularity corpuscular 0.40 ± 0.03 vs. plasma groups 0.30 ± 0.03, *p* < 0.05).

**FIG. 8.**
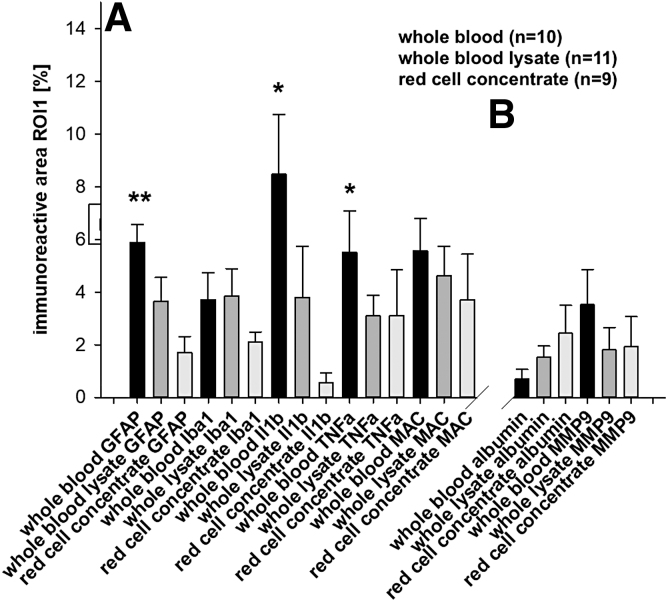
Inflammatory reaction (**A**) and BBB function (**B**) in corpuscular subgroups. In the whole blood group, there was higher immunoreactivity in GFAP, IL1b, and TNF-α. There were no differences in BBB function between groups; *p < 0.05 versus red cell concentrate, **p < 0.05 versus whole blood lysate and red cell concentrate. Values are given as mean ± SEM. BBB, blood–brain barrier; GFAP, glial fibrillary acidic protein; Iba1, ionized calcium-binding adaptor molecule 1; IL1b, interleukin-1ß; MAC, membrane attack complex; MMP9, matrix metalloproteinase 9; SEM, standard error of the mean; TNF-α, tumor necrosis factor alpha.

In a subgroup analysis of the corpuscular groups, there was significant GFAP immunoreactivity in the whole blood group (whole blood 5.9 ± 0.7, whole blood lysate 3.6 ± 0.9%, and red cell concentrate 1.7 ± 0.6%, *p* < 0.05 whole blood vs. whole blood lysate and red cell concentrate). Further, there was more reactivity in IL1b (whole blood 8.5 ± 2.3, whole blood lysate 3.8 ± 2.0, and red cell concentrate 0.6 ± 0.4%, *p* < 0.05 whole blood vs. red cell concentrate) and TNF-α (whole blood 5.5 ± 1.6, whole blood lysate 3.1 ± 0.8, and red cell concentrate 1.0 ± 0.6%, *p* < 0.05 whole blood vs. red cell concentrate; Fig. 8). However, there were no differences in Iba1 and MAC immunoreactivity and microglia morphology.

### Blood–brain barrier

Overall, significant signs of BBB breakdown were observed, First, we observed trans-BBB albumin leakage in the corpuscular group (albumin corpuscular 1.1 ± 0.3 vs. plasmatic 0.6 ± 0.3%, *p* < 0.001; Fig. 6, right panel). Further, MMP9 was increased as a sign of loss of barrier integrity in the corpuscular groups (MMP9 corpuscular 2.7 ± 0.7 vs. plasmatic 0.3 ± 0.1%, *p* < 0.05; Fig. 6, right panel). When analyzing the corpuscular subgroups, we found no differences between groups (Fig. 8, right panel).

## Discussion

Surgical evacuation is frequently considered necessary for traumatic ASDHs; however, ongoing debates persist regarding the optimal surgical approach.^20^ When compared to chronic subdural hematoma, there are little data available on medical treatment^21^ and basic research on the influence of different blood components on brain injury after subdural hematoma is scarce. One study has specifically explored the variances in secondary ischemic lesions induced by various blood components in the subdural space using rats. Subsequently, blood constituents were shown to influence the extent of ischemia beneath the subdural heomrrhage.^22^ The current study therefore aimed to explore the diverse pathophysiological factors associated with blood components in ASDH.

### Cerebral blood flow and blood–brain barrier

Studies from ICH show that the combination of ICP and blood-derived vasoactive substances seem to influence CBF.^23^ This may be, at least partially, attributable to toxic effects of blood cells and their constituents. Lysed red blood cells led to 3-fold increased edema formation after ICH in a rat model, suggesting that intracellular components are harmful.^24^ For example, the release of CA-I from lysed red blood cells mediates an increase in the permeability of the BBB.^25^ Moreover, Hb itself has been shown to have a toxic effect on neurons through an iron-dependent, oxidative mechanism.^26^ Further, Hb has various supposed vasoconstrictive properties, including induction of an increased production of vasoconstrictive prostaglandins and lipid peroxides.^27^ However, a superficial layer of blood on the cortex without raised ICP is insufficient to cause significant brain damage.^28^

Whereas local toxic effects of various blood components have been demonstrated, the elevation of ICP caused by silicone oil is inadequate to induce massive neuronal damage.^29^ A rat model showed that subdural blood, both venous and arterial blood, aggravates brain edema and lesion development compared to subdural saline infusion.^22^ Further, short-lasting hippocampal hypermetabolism and reduced blood flow were observed after subdural blood infusion, but not after subdural silicone gel mass insertion, suggesting that blood constituents may be responsible for excitotoxicity.^30^ After blood infusion, hippocampal damage was not observed after between 8 and 24 h, but after 5 days, some hippocampal neurons demonstrated structural changes.^30,31^ In addition, the different subdural distribution of infused fluids may have led to other local effects. Studies using silicone oil with viscosity comparable to blood showed similar intracranial distribution; however, ischemic damage was less pronounced and subdural artificial cerebrospinal fluid infusion caused no harm at all.^23^ It has therefore been proposed that both tissue pressure and vasoactive substances are components of the immediate reduction in blood flow after ICH.^31^

In the current study, we observed that the sum of lesion volumes of the red cell concentrate and plasma groups (0.60 ± 0.09 mm^3^) still only constituted a fraction of the lesion volume in the whole blood group (21.3 ± 6.5 mm^3^). There were more pronounced effects of whole blood infusion on CBF, supporting the strategy of early operative removal of a subdural hematoma in patients to prevent such focal effects. Admittedly, CBF was only monitored in a single location in our study. In previous studies with measurement of contralateral focal CBF, we found that ASDH causes CBF reduction with a large radius.^4^ Nevertheless, laser scanning for the assessment of regional CBF would have been more accurate.^32^ Further, a possible protective component of plasma must be considered. According to a TBI/hemorrhagic shock study with fresh-frozen plasma resuscitation, there is a beneficial effect on the BBB and lesion size beyond the volume expander effect, suggesting that plasma possesses biological properties beneficial for injured tissues.^33^ In our study, MMP 9 levels as a marker for late BBB disruption^34^ were low in the plasmatic, but not in the corpuscular, group. Consequently, bevacizumab emerges as a potential early targeted therapy for traumatic brain edema. The optimal treatment window for bevacizumab in traumatic brain edema within a rat model is within 12 h post-TBI, with 1 h identified as the optimal therapeutic time point.^35^

### Thrombin

Among the corpuscular groups, the red cell concentrate group suffered minor lesions. This indirectly points toward the role of thrombin as a factor in secondary damage. Aside from the effects of blood constituents on CBF, direct contact of thrombin with neurons can induce cell death in a dose-dependent manner,^13^ with higher amounts of thrombin causing neuroinflammation and apoptosis.^36^ A possible mechanism may be excitotoxicity with increased glutamate efflux,^37^ which may contribute to spreading ischemia.^38^ We therefore attempted to elucidate the effect of thrombin by infusing blood plasma and anticoagulated blood plasma in the subdural space. Evidence shows that intracerebral infusion of blood and paraffin both cause upregulation of the local cerebral thrombin system independently of whole blood constituents and that neuronal loss is associated with the amount of local thrombin expression.^39^ A previous study of ASDH and protease-activated receptor 1 blockage demonstrated tendentially smaller lesion volumes. Despite these findings, the small amount of thrombin within the subdural blood seems insufficient to cause damage or perhaps was insufficient to permeate into the parenchyma.^15^ Moreover, CBF was not affected in the plasmatic infusion groups, indicating that the pressure-associated local thrombin system may not have been upregulated in our study, as is observed in ICH.^39^

### Intracellular factors and inflammatory reaction

Both whole blood groups had the most significant deficits and lesions, but the whole blood lysate group showed fewer inflammatory reactions. This may possibly be attributed to a hemolysis-associated loss of (intracellular) fluid and therefore less sustained ICH. A further possible explanation may be that protective intracellular factors, such as glutathione/glutathione peroxidase,^40,41^ could promote the smaller lesion size and inflammatory response in the whole blood lysate group compared with the whole blood group.

Oxidative damage reactions are well-validated mechanisms for secondary injury in TBI models.^42^ High levels of cytoplasmic antioxidants are found in erythrocytes, and their coordinated actions protect erythrocytes from free radicals. The main enzyme components of the antioxidant system of erythrocytes are superoxide dismutase (SOD) and catalase (CAT).^43^ In the laboratory setting, scavenging of anions with SOD has been shown to be beneficial in several types of traumatic and ischemic injury^44^; however, whole blood lysate lesions were still relatively high. As such, it was proposed that intracellular substances, such as Hb,^27^ cathepsin B,^45^ CA-I,^25^ or calpains,^46^ could be causing additional damage. Moreover, products of hemolysis in the subarachnoid space with high K^+^-content are known to cause delayed ischemic deficits.^10^

The proposed pathophysiological correlate for these findings is cortical spreading depolarizations (CSDs), which can cause vasoconstriction, commonly known as spreading ischemia.^47,48^ Whereas no CSDs were found to occur in response to irrigation with K^+^ at 35- and 50-mmol/L concentrations, they do appear at ≥80-mmol/L levels.^10^ Electrochemical failure caused by CSDs seems more deleterious when accompanied by low perfusion.^47^ Spreading ischemia can also be induced by blood-derived oxyhemoglobin combined with the vasoconstrictor, endothelin-1 (ET-1).^49^ Minimally invasive clot evacuation has therefore been found to decrease ET-1 levels and subsequent BBB permeability.^50^ Although the extent to which CSDs influenced lesion size was not part of the current study, we have previously shown that CSDs occur in the same ASDH model and promote secondary damage.^51^ However, K^+^ concentrations in the lysed whole blood used in the current study were 42.0 ± 0.9 mmol/L, which might be below the threshold for causing spreading ischemia.^10^

Overall, there seems to be a coexistence of harmful and protective intracellular substances in erythrocytes with an advantage being noted for anti-inflammatory reactions in our study. There was significantly increased inflammation displayed by increased immunoreaction of IL-1ß and TNF-α after whole blood infusion. As previously reported in TBI in rats^52^ and humans,^53^ we also found increased perilesional MAC immunoreactivity in the corpuscular group. Activation of the complement cascade and formation of MAC is most likely to begin within 48 h after injury.^52^ In the current study, there were no differences in Iba1 immunoreactivity pointing toward microglia proliferation. Pronounced microglial activation^54^ after subdural whole blood infusion could be identified by morphological analysis. Although a previous study observed increased GFAP and Iba1 immunoreactivity 96 h after ASDH, these changes were not observed after 24 h.^9^

### Limitations

In the context of multiple blood constituents, the current study was unable to identify a singular factor which was predominantly accountable for the secondary injury. The examination of larger overall lesion volumes, particularly in the plasmatic group, could have clarified the impact of plasmatic components, such as thrombin. Regarding inflammation, the timing of measurement at 48 h post-ASDH might have been too early. Additionally, the distribution of constituents likely varied because of their viscosity, which was not investigated in the study.

## Conclusion

In our study, no single factor could be found solely responsible for the observed damage, but rather the combination of different pathophysiological factors lead to injury.

There were more pronounced effects of whole blood infusion on CBF, underscoring the role of early surgical removal of subdural hematoma to prevent such focal mechanical effects. Further, we identified the pronounced inflammatory reaction in ASDH as a target for further research with immunosuppressants such as cyclosporine or ethyl pyruvate.^55,56^ Pronounced BBB disruption may warrant the investigation of medication such as bevacizumab^35^ against edema formation or novel dynamic platforms for recapitulating BBB function *in vitro*, screening potential novel therapeutics, and establishing a relevant paradigm to evaluate the pathophysiology of TBI.^57^

## Abbreviations Used

ASDH: acute subdural hematoma
BBB: blood–brain barrier
CA-1: carbonic anhydrase 1
CBF: cerebral blood flow
CSDs: cortical spreading depolarizations
ET-1: endothelin-1
GFAP: glial fibrillary acidic protein
Hb: hemoglobin
Iba1: ionized calcium-binding adaptor molecule 1
ICH: intracranial hemorrhage
ICP: intracranial pressure
MAC: membrane attack complex
MMP9: matrix metalloproteinase 9
ROI: region of interest
SEM: standard error of the mean
SOD: superoxide dismutase
TBI: traumatic brain injury
TNF-α: tumor necrosis factor alpha
